# Uncovering the Role of Small and Intermediate-Length Non-Coding RNAs in Glioma Pathogenesis and Prognosis

**DOI:** 10.3390/cancers18193232

**Published:** 2026-10-08

**Authors:** Jasmine Machhi, Priya Kumthekar, Stella Hartmann

**Affiliations:** 1 Division of Neurology (Neuro-Oncology), Northwestern University Feinberg School of Medicine, Chicago, IL 60611, USA; 2 Belay Diagnostics LLC, Chicago, IL 60607, USA

**Keywords:** small non-coding RNA, intermediate-sized non-coding RNA, glioma, glioblastoma, tumorigenesis, cancer

## Abstract

Gliomas are the most prevalent and aggressive primary tumors of the central nervous system (CNS), with glioblastoma demonstrating particularly poor survival rates despite multimodal treatment approaches. The extensive molecular heterogeneity and resistance to conventional therapies highlight the urgent need for novel biomarkers and therapeutic targets. Non-coding RNAs (ncRNAs), transcripts that do not encode proteins, have emerged as crucial regulators of gene expression and cellular homeostasis in both normal and malignant contexts. Their dysregulation contributes to key oncogenic processes, including uncontrolled proliferation, apoptosis, evasion, invasion, and therapeutic resistance. Small and intermediate-sized are strong candidates for biomarkers due to their high abundance and relative stability. Previous reviews on ncRNAs highlight the numerous roles of microRNAs, long non-coding RNAs (lncRNAs), and circular RNAs (circRNAs) in gliomas. Here, we focus specifically on small and intermediate-length non-coding RNAs, including transfer RNAs (tRNAs) and tRNA-derived fragments (tRFs or tsRNAs), small nucleolar RNAs (snoRNAs), vault RNAs (vRNAs or vtRNAs), small nuclear RNAs (snRNAs) and mitochondrial tRNAs (mt-tRNAs). Recent studies suggest that these various non-coding RNAs participate in diverse aspects of glioma biology, including regulation of gene expression and modulation of metabolic and immune pathways, and correlate with tumor grade and molecular subtype. In this review, we summarize current evidence regarding the expression profiles and functional roles of small and intermediate-length non-coding RNAs in glioma tumorigenesis, progression, and therapeutic response. The differential expression of many of these ncRNAs in gliomas implies their involvement in both tumor-suppressive and oncogenic processes. A deeper understanding of these molecules may provide valuable insights into glioma biology and reveal novel biomarkers for diagnosis, prognosis, and treatment.

## Simple Summary

Gliomas are aggressive brain tumors that remain difficult to treat and are often associated with poor patient outcomes. A major challenge in their management is their pronounced molecular complexity, which hinders the identification of reliable diagnostic markers and predictors of treatment response. In recent years, it has become clear that non-coding RNAs (ncRNAs) play critical roles in cancer biology. Although prior reviews have examined the roles of microRNAs (miRNAs), long non-coding RNAs (lncRNAs), and circular RNAs (circRNAs) in gliomas, other small- and intermediate-length ncRNA types remain comparatively underexplored. This review focuses on those ncRNAs, including transfer RNAs (tRNAs) and tRNA-derived fragments (tRFs or tsRNAs), small nucleolar RNAs (snoRNAs), vault RNAs (vRNAs or vtRNAs), small nuclear RNAs (snRNAs) and mitochondrial tRNAs (mt-tRNAs). By integrating recent findings, this review highlights the potential of understudied non-coding RNAs as novel biomarkers and therapeutic targets, offering new perspectives for future brain tumor research and clinical translation.

## 1. Introduction

Gliomas represent the most prevalent and aggressive primary tumors of the central nervous system. Glioblastoma, the most aggressive subtype, exhibits a particularly poor survival rate, with a 5-year survival rate of 6.9% [1]. Mainstay treatment modalities for patients with glioblastoma include maximal surgical resection, followed by radiotherapy, and chemotherapy with the drug temozolomide [2]. Despite ongoing advances in these treatment regimens they remain insufficient due to glioblastoma exhibiting extensive molecular heterogeneity and intrinsic resistance to these conventional therapies [3]. This underscores the urgent need for novel biomarkers and therapeutic targets to improve clinical outcomes in glioma patients.

Emerging research has identified non-coding RNAs (ncRNAs) as critical regulators of gene expression and cellular function across various physiological and pathological contexts, including gliomas [4,5]. These molecules modulate key biological processes such as cell proliferation, migration, apoptosis, immune evasion, and therapeutic resistance [4,5,6]. Many ncRNAs exhibit pro-oncogenic or tumor-suppressive functions, and their dysregulation has been closely linked to glioma initiation, progression, and recurrence [4,5,6]. Their expression patterns in tumor tissue have revealed diagnostic, prognostic, and therapeutic potential, positioning ncRNAs as promising molecular biomarkers in neuro-oncology.

Several properties of ncRNAs make them great targets for biomarkers. ncRNAs are more abundant than messenger RNAs (mRNAs), with estimates suggesting that over 90% of the human genome is transcribed into ncRNAs, while less than 2% encodes mRNAs for protein synthesis [7,8]. This higher abundance may enhance assay sensitivity by providing more readily detectable targets, while changes in their abundance may more reliably reflect disease states. The abundance of ncRNAs also reflects their stability. ncRNAs are highly stable due to their intrinsic structures. For example, tRNAs and rRNAs contain many secondary and tertiary elements that shield their backbones from degradation by endonucleases and exonucleases [9,10]. Many ncRNAs contain certain modifications and can be bound to RNA-binding proteins, also enhancing their stabilities [11]. Their packaging into extracellular vesicles also help shield them from extracellular ribonucleases (RNases) [12,13]. Taken all together, the enhanced stability further supports their utility as a potential biomarker as they would be a more reliable and abundant target.

Other review articles on ncRNAs specifically highlight the roles of microRNAs (miRNAs), long non-coding RNAs (lncRNAs), and circular RNAs (circRNAs) in gliomas [5,14,15,16,17]. They have shown that these classes are involved in glioma tumorigenesis, tumor growth, and resistance to chemotherapy. By consolidating the current literature of other non-coding RNAs, we aim to deepen understanding of their mechanistic roles in glioma tumorigenesis and to identify potential biomarkers for diagnosis, prognosis, and therapeutic targeting. Collectively, the literature demonstrates how ncRNAs have utility in potential biomarkers for diagnosis and prognosis as well as potential treatment targets.

## 2. tRNAs

Transfer RNAs (tRNAs), which range from 70 to 100 nucleotides (nt), are essential components of the cellular translational machinery. Recent findings have significantly expanded the various roles of tRNAs, especially with respect to how they may contribute to glioma progression. This is often mediated by post-transcriptional modifications that alter tRNA structure, stability, and function, giving rise to altered translation dynamics and novel regulatory RNA fragments that contribute to tumor progression and changes in the tumor microenvironment [18].

In gliomas, specific tRNA modifications have been shown to directly impact tumorigenic processes. The methyltransferases TRMT10A, TRMT10B, and TRMT10C, which install m^1^G9 modifications on tRNA-ArgCCT and tRNA-Trp, play a critical role in maintaining tRNA stability. Downregulation of TRMT10A in glioma cells reduces m^1^G9 levels, destabilizes tRNA-ArgCCT and tRNA-Trp, and promotes the generation of the tRNA-derived fragment tRF-22. This fragment represses MXD1, a transcriptional repressor of HIF1A, thereby enhancing HIF1A-driven vasculogenic mimicry (VM) which is a hallmark of aggressive glioblastoma [19]. Overexpression of TRMT10A mitigates tumor growth and VM in mouse models, emphasizing the relevance of m^1^G9 modifications on tRNA-ArgCCT as a modulator for glioblastoma progression (Table 1) [19].

Another modification of tRNAs involves m^7^G (N7-methylguanosine). Regulators of this methylation modification have been shown to be more highly expressed in glioma tissues compared to normal tissues [30,31]. One such regulator includes the METTL1/WDR4 complex. Overexpression of METTL1 enhances m^7^G levels and boosts the abundance of specific tRNAs, such as Arg-TCT-4-1 [32]. This tRNA then promotes the translation of AGA-rich mRNAs involved in cell cycle progression [32]. A study specifically found that METTL1 was amplified in 13% of glioblastoma patients and correlates with poor patient outcomes [32]. The expression of METTL1 was found to be significantly higher in low-grade glioma and glioblastoma tissues compared to normal tissue samples [33]. In glioma, its high expression was also associated with poor prognosis, specifically in patients with gliomas of WHO Grade II, astrocytoma, and IDH-wildtype tumors [33]. Moreover, elevated m^7^G scores are linked with higher tumor mutation burden and immune activation, suggesting a role for METTL1 not only in driving proliferation but also in shaping the immunological landscape of the tumor [33,34].

Another key modification involves pseudouridylation, a chemical modification of RNAs where a uridine base is converted into a structure isomer pseudouridine (Ψ). Pseudouridylation can affect RNA–protein interactions, influencing RNA processing, localization, stability, and translation [35]. Pseudouridine is the most abundant RNA modification, and increased levels of pseudouridine have been detected in patients with cancer, specifically gliomas [35,36]. The expression of multiple pseudouridine synthase (PUS) enzymes has implications on median survival of glioma patients. Specifically, the expression of enzyme Pseudouridine Synthase 7 (PUS7), which modifies tRNAs (among other RNAs), has been found to be significantly upregulated in glioblastoma patients compared to non-tumor controls and was also correlated with worse patient survival [36]. Lower expression of PUS7 in this subtype was found to have improved patient survival [36]. Mechanistically, PUS7-mediated tRNA modifications lead to a reduction in TYK2 translation and downregulation of the interferon-STAT1 signaling pathway, which normally acts to restrict tumor proliferation [37]. These findings suggest that PUS7 promotes glioma progression by altering the translational landscape and decreasing anti-proliferative immune signaling.

Another modification is t^6^A (N6-threonylcarbamoyladenosine), which occurs on ANN-decoding tRNAs and drives high translation rates in glioma stem cells (GSCs). This modification is catalyzed by the enzyme YRDC, which is facilitated by threonine accumulation. The enhanced t^6^A modification supports ANN codon-dependent translational reprogramming, driving cell cycle progression and tumor growth. Notably, depletion of YRDC or restriction of threonine intake significantly reduces t^6^A levels, leading to decreased translation efficiency, impaired GSC survival, and suppressed glioblastoma progression. Clinically, elevated YRDC expression correlates with high translational activity in glioblastoma tumors, underscoring its role in promoting malignancy [38].

tRNA levels themselves also appear to be deregulated in gliomas (Table 1). Macari et al. found that both initiator methionine (tRNAiMet) and elongator methionine tRNA (tRNAeMet) expression were elevated in glioblastoma tissues compared to grade II/III glioma tissue (Table 1) [23]. Yang et al. found that tRNAiMet may function as a growth-supporting RNA in glioblastoma cells: CRISPR/Cas9-mediated knockdown of tRNAiMet markedly inhibited growth and proliferation, both in the U251 glioma cell line and in primary glioma stem cell isolates, while ectopic expression of tRNAiMet partially rescued the proliferation lost with SOX4-mediated repression [39].

Furthermore, RNA methyltransferase Nop2/Sun RNA methyltransferase (NSUN6) has been implicated in glioma through its role in RNA 5-methylcytosine (5mC) modification and response to temozolomide (TMZ) [40]. In glioblastoma, high NSUN6 expression was associated with longer survival across different The Cancer Genome Atlas (TCGA) data sets, and this association was reported to be independent of MGMT promotor methylation status [40]. Functional studies further showed that NSUN6-mediated RNA 5mC modification influences glioblastoma cellular responses to TMZ through regulation of NELFB and RPS6KB2 [40]. These findings suggest that NSUN6 may have both prognostic and therapeutic relevance in glioblastoma; however, the reported survival association is based on retrospective TCGA analyses and has not been established as a clinically validated prognostic biomarker.

Collectively, these findings show that tRNAs and their modifications are dynamic regulators of gene expression, metabolic reprogramming, and immune evasion in gliomas. The dysregulation of tRNA-modifying enzymes and the resulting changes in tRNA structure and function not only promote glioma progression but may also offer prognostic value in survival outcomes of patients with gliomas.

## 3. tRNA-Derived Fragments

tRNA-derived fragments (tRFs), also known as tRNA-derived small RNAs (tsRNAs), have emerged as important regulatory molecules in many cellular processes. These fragments are generated from specific cleavage of mature or precursor tRNAs, ranging from 14 to 50 nt, and are increasingly recognized for their roles in tumor progression, cell signaling, and extracellular communication [41]. Importantly, tsRNAs have also been found to play a role in gliomas and may serve as a biomarker for tumor subtypes [42].

In a recent study involving four glioblastoma and five low-grade glioma patients, high-throughput sequencing identified 186 tsRNAs across the two groups, with 43 tsRNAs unique to glioblastoma and 24 specific to low-grade glioma [25]. Among these, nine candidate tsRNAs were selected based on expression patterns; six were highly expressed in glioblastoma and three were expressed at lower levels. qPCR validation confirmed that five tsRNAs were significantly upregulated in glioblastoma, and one was significantly downregulated in glioblastoma samples. The upregulated tsRNAs include tiRNA-1-33-Gly-GCC-1, tiRNA-1-33-Gly-CCC-1, tRF-1-31-His-GTG-1, tiRNA-1-33-Gly-GCC-2-M3, and tiRNA-1-34-Lys-CTT-1-M2 (Table 1). These tsRNAs were associated with various cancer-related pathways such as axon guidance, Hippo signaling, and regulation of pluripotent stem cells [25]. In contrast, the downregulated tsRNA, tRF-1-32-chrM.Lys-TTT, was linked to oocyte meiosis and renin secretion (Table 1) [25]. The following three tsRNAs were found to have no significant difference in expression between glioblastoma and low-grade glioma: tRF-1-22-chrM.Ser-GCT, tiRNA-1-34-Lys-CTT-3, and tRF-+1-T17-Ser-TGA-1 [25].

In another study that compared low-grade astrocytoma tissues and non-tumor controls, the expression levels of four tsRNAs, tRFdb-3003a, tRFdb-3003b, ts-55, and ts-60, were found to be significantly downregulated in tumor samples (all *p* < 0.05) (Table 1) [26]. Two additional tsRNAs, tRFdb-5017a and tRFdb-5017b, did not have a significant difference between low-grade astrocytoma tissue and non-tumor tissues (Table 1) [26]. These consistent downregulations suggest that specific tsRNAs may act as tumor suppressors and that their loss could contribute to glioma pathogenesis, especially in astrocytoma.

An additional study looking at expression of tsRNAs in low-grade glioma and glioblastoma tissue samples found that two tsRNAs, tRF-30-87R8WP9N1EWJ and tRF-30-PNR8YP9LON4V, were the most abundant in these samples (Table 1) [28].

Neither tsRNA showed a significant difference in expression between the low-grade glioma and glioblastoma groups; however, both were significantly upregulated in U87, U251, and U373 glioma cell lines compared with human astrocytes (Table 1) [28]. This finding suggests that tsRNAs may be associated with glioma cells but do not distinguish between tumor grades.

tsRNAs also appear in the extracellular RNA landscape in gliomas, particularly within exosomes. In a study analyzing plasma-derived exosomes from glioma patients, a total of 453 differentially expressed tsRNAs were identified, with 119 upregulated and 334 downregulated, highlighting a widespread alteration in tsRNA profiles associated with glioma pathology [24]. Among these, i-tRF-LeuCAA was significantly elevated in plasma-derived exosomes from glioma patients compared with healthy controls and was associated with poorer overall survival (Table 1) [24]. These findings identify i-tRF-LeuCAA as a candidate diagnostic and prognostic biomarker; however, independent and prospective clinical validation was required to establish its clinical utility. Liquid biopsy studies further highlight the potential diagnostic and prognostic relevance of tsRNAs in gliomas. In CSF samples, tsRNAs made up over 30% of differentially expressed cell-free RNAs (cfRNAs) between glioma patients and tumor-free central nervous system disease patients, placing them among the most prominent RNA species expressed alongside mRNAs [27]. Specifically, multiple tRFs, including tRF-20-R959KUMK, tRF-26-3K7SIR3DR20, tRF-30-F1R3WE8RO86J, tRF-32-FZ9KR959NRZZP, tRF-34-M2OSRNLNKSEKH9, tRF-35-QKF1R3WE8RO86J, tRF-37-QB1MK8YUBS68BFJ, tRF-39-J9ZH0SR9593KMHFV, tRF-22-R3WE8RO84, and tRF-16-Y9H33PD, were significantly upregulated while tRF-17-8SOUPR2 was significantly downregulated in the CSF of glioma patients compared with tumor-free controls (Table 1) [27]. Notably, several tsRNAs derived from AspGTC tRNAs (amino acid aspartic acid with an anticodon of GTC) were found to be upregulated in the CSF of brain tumor patients, with a conserved sequence motif suggesting common functional roles across tumor samples [27]. tsRNAs also comprised 21% of differentially expressed cfRNAs in the plasma of glioma patients compared with controls; however, fewer individual tsRNAs demonstrated significant differential expression in plasma, with tRF-26-R38E8RO86JD being upregulated while tRF-16-8R6546D, tRF-18-HRMFWRV, tRF-26-R3WE8RO86JD, tRF-28-08R959KUMK9, and tRF-35-DEU2O1B54ZUPX1 showed no significant differences between groups (Table 1) [27]. Together, these findings demonstrate that tsRNAs were differentially expressed in glioma cfRNA, particularly in CSF, supporting their potential utility as minimally invasive biomarkers for glioma detection [27].

## 4. snoRNAs

Small nucleolar RNAs (snoRNAs) are a class of non-coding RNAs found in the nucleolus of eukaryotic cells [43]. snoRNAs have a length of 60-300 nt and play a role in ribosomal processing, specifically guiding chemical modifications of RNA [43]. Based on their structure and function, snoRNAs are classified into C/D box snoRNAs (SNORDs) and H/ACA box snoRNAs (SNORAs). SNORDs primarily guide the 2′-O-methylation of rRNA in the nucleolus, and SNORAs direct the pseudouridylation of rRNA and snRNA [44,45]. Another specialized class of snoRNAs, small cajal body-specific RNAs (scaRNAs), localize specifically to Cajal bodies. Unlike other snoRNAs that accumulate in the nucleolus, scaRNAs contain a specific Cajal body-specific localization signal, known as the CAB box, which was required for their retention in Cajal bodies [46]. They often contain box C/D and H/ACA domains, sometimes as chimeric molecules, which guide modification and process rRNA and snRNAs [46]. Beyond these roles in RNA functioning and modification, snoRNAs have also been found to play a significant role in many tumor types, especially gliomas (Table 2).

Differential expression of snoRNAs and scaRNAs was observed in pediatric diffuse intrinsic pontine glioma (DIPG) tissues compared with controls and pediatric non-brainstem astrocytoma [20]. Relative to controls, 41 snoRNAs were significantly upregulated, and 48 were downregulated in DIPG, whereas comparison with pediatric non-brainstem astrocytoma identified 13 upregulated and 109 downregulated snoRNAs [20]. For scaRNAs, five were upregulated and none were downregulated in DIPG compared with controls; conversely, five scaRNAs were downregulated and none were upregulated in DIPG compared with pediatric non-brainstem astrocytoma [20]. The snoRNAs and scaRNAs with the greatest increases in DIPG relative to controls included SNORA38B, SCARNA4, SNORA31, SCARNA8, SNORA2A, SNORA54, SNORA10, SNORD92, and SNORD53, whereas the most strongly downregulated transcripts included SNORD115-44, SNORD115-15, SNORD115-17, SNORD115-18, SNORD115-19, SNORD115-31, SNORD115-30, SNORD115-27, SNORD115-25, SNORD115-32, SNORD115-10, and SNORD115-24 (Table 2) [20]. Compared with pediatric non-brainstem astrocytoma, the most highly upregulated snoRNAs in DIPG were SNORA54, SNORA11E, SNORD113-7, and SNORD114-5, while SNORD91B, SNORD115-15, SNORD115-10, SNORD115-42, SNORD115-6, SNORD115-22, SNORD115-29, SNORD115-36, and SNORD41 showed the greatest downregulation (Table 2) [20].

Many studies have also focused on specific snoRNAs and their various roles in the tumorigenesis, prognosis, and staging of gliomas. SNORD12B was significantly upregulated in glioblastoma tissues and cell lines and was expressed at higher levels in WHO grade III–IV gliomas than in WHO grade I–II tumors (Table 2) [49]. Mechanistically, SNORD12B promotes glioblastoma progression by enhancing glycolipid metabolism and cellular proliferation through the MSI2–SNORD12B–FIP1L1–ZBTB4 regulatory feedback loop [49]. Knockdown of SNORD12B suppresses glycolipid metabolism and proliferation, thereby inhibiting tumor growth and highlighting SNORD12B as a potential oncogenic driver [49]. In contrast, SNORD47 (RUNU47/U47) was downregulated in surgically resected glioma tissues compared to adjacent non-tumor tissues, with significantly greater downregulation in WHO grade IV tumors than in grade III and grade I/II; no significant difference was observed between grade I/II and grade III tumors [51]. Functionally, SNORD47 overexpression inhibited glioma cell proliferation, colony formation, migration, and invasion while inducing G2-phase cell-cycle arrest. Moreover, SNORD47 overexpression enhanced the sensitivity of glioma cells to TMZ, resulting in greater inhibition of proliferation and increased G2-phase arrest compared with either treatment alone. These findings suggest a potential synergistic interaction between SNORD47 and TMZ and support a tumor-suppressive role for SNORD47 in glioma [51].

Similarly, the expression of SNORD76 (U76) was downregulated in surgically resected glioblastoma (WHO grade IV) tissue samples compared to grade II and grade III gliomas, but there was no significant difference between grade II and grade III (Table 2) [52,53]. Specifically, lower SNORD76 expression was associated with shorter overall survival based on these findings. Li et al. concluded SNORD76 might be a potential prognostic biomarker (Table 2) [52]. SNORD76 contributes to repressed proliferation and growth of glioma cells through its effect on cyclinD1 and p21 [52]. SNORD44 was also downregulated in glioma tissues compared to normal tissues, and lower expression of SNORD44 was associated with a higher WHO grade (Table 2) [50]. Based on in vitro studies with glioma cell lines the overexpression of SNORD44 suppresses glioma cell growth, invasion, and migration [50]. Conversely human snoRNA-jouvence (h-jou) has been shown to have low expression in the U87 glioblastoma cell line, and its low expression was associated with decreased cell proliferation and higher apoptosis of glioblastoma cells (Table 2) [47,55].

In glioblastoma, the U3 snoRNA (RUNU3) has also been implicated in tumor metabolism and growth [54]. U3 expression was significantly elevated in IDH1 wild-type glioblastoma compared to mutant forms and normal brain tissue (Table 2) [54]. Mechanistically, U3 enhances aerobic glycolysis and proliferation through the U3/ZBTB7A/HK2/LDHA axis [54].

In LGG, snoRNA expression patterns have also been investigated as candidate prognostic signatures A seven-snoRNA signature comprising of SNORA32, SNORA36B, SCARNA15, SNORD88C, SNORD38A, SNORA63D, and SNORA63E was developed using Cox regression and LASSO modeling in a retrospective TCGA-LGG cohort of 476 patients [56]. The cohort was randomly divided into a training set (n = 333) and an internal validation set (n = 143), and the resulting signature demonstrated prognostic performance in the reported validation cohort [56]. Several components of the signature, including high tissue expression of SNORA32, SNORA36B, SCARNA15, SNORD88C, and SNORD38A, were associated with poorer outcomes, whereas SNORA63E was downregulated in the high-risk group, and SNORA63D had no significant difference between high- and low-risk LGG groups (Table 3) [56]. The signature retained prognostic performance across WHO grade II and grade III subgroups and IDH mutation status [56]. These findings support the potential prognostic value of the signature, although validation in prospective clinical studies will be required to establish its generalizability and clinical utility. Among its components, SNORD88C has been functionally investigated in vitro, where knockdown reduced glioma cell proliferation, invasion, and migration [56]. An additional 11-snoRNA signature was developed to investigate the prognostic significance of snoRNAs in LGG [52]. Following exclusion of recurrent tumors and patients with missing or zero survival time, a history of other malignancies, or prior radiotherapy or chemotherapy, 488 patients from the TCGA-LGG cohort were included in the subsequent survival and functional mechanism analyses [52]. Deng et al. identified 21 snoRNAs as significantly associated with overall survival outcomes of LGG, including 11 snoRNAs associated with poorer prognosis, namely, SNORA40, SNORD 115-45, ACA59, SNORD73B, SNORA63, SNORA31, SNORA80D, SNORA72, U3 217-127, SNORD91A, and SNORA22B (Table 3) [57]. Of the 11, ACA59, SNORA22B SNORA63, SNORA72, and SNORD91A were upregulated in high-risk LGG vs. low-risk LGG, while SNORA31, SNORA40, SNORD73B, SNORA80D, SNORD115-45, and U3 217-127 were downregulated in high-risk LGG vs. low-risk LGG (Table 3) [57]. Further gene set enrichment and functional analyses indicated that this snoRNA signature was associated with key oncogenic signaling pathways and biological processes, including the cell cycle, Wnt, MAPK, JAK/STAT, T cell receptor, and NF-κB signaling [57]. While these findings support an association between snoRNA expression and patient outcome, they were based on retrospective cohort analysis and should therefore be considered candidate prognostic associations rather than clinically validated biomarkers. Independent and prospective validation will be required to establish their clinical utility.

SNORA71B expression was significantly higher in glioma tissues than normal brain tissue and was positively associated with tumor grade (Table 2) [48]. Higher SNORA71B expression has also been associated with shorter overall survival and disease-free survival (Table 2) [48]. Functional studies in glioblastoma cell lines suggest a potential role in malignant phenotypes as SNORA71B knockout reduced cell viability, migration, and invasion (Table 2) [48]. However, these findings remain based on retrospective tissue-level associations and experimental models, and clinical validation of SNORA71B as a prognostic biomarker is lacking.

In a study by Huang et al., liquid biopsy analysis comparing plasma cfRNA from glioma patients and healthy controls demonstrated differential expression of some, but not all, snoRNAs. Specifically, SNORD104 was significantly downregulated in glioma patients while SNORD38A showed no significant difference in expression between the two groups (Table 2) [27].

Modifications of snoRNAs by DKC1, a pseuduridine synthase, have also been associated with glioma prognosis. Higher DKC1 expression in tissue samples of LGG patients was associated with poor survival [54]. However, this association is based on retrospective analysis and has not been clinically validated as a prognostic biomarker in glioma [58].

In pediatric high-grade gliomas (pHGGs), TP53 and H3F3A mutations have been linked to distinct snoRNA expression patterns, highlighting a potential role for snoRNAs in tumor pathogenesis [59]. There is limited overlap in commonly mutated genes, including TP53, H3F3A and IDH1, between pediatric and adult glioblastoma subtypes, underscoring fundamental differences in their molecular landscapes and disease mechanisms [60,61]. Comparative analysis of snoRNA expression in H3F3A-mutant and wild-type (WT) pediatric glioblastoma relative to pediatric non-tumor control tissues identified distinct deregulation patterns (Table 4) [59]. In the H3F3A-mutant subgroup, 65 snoRNAs were differentially expressed, including 63 upregulated and two downregulated transcripts (Table 4) [59]. In contrast, the H3F3A-WT subgroup exhibited differential expression of 104 snoRNAs, of which 68 were upregulated and 36 downregulated (Table 4) [59]. Notably, 48 snoRNAs (46 upregulated and two downregulated) were shared between TP53-mutant and TP53-WT subsets. The HBII-52 snoRNA cluster was specifically downregulated in H3F3A-WT tumors (Table 4) [59]. Stratification by TP53 status further revealed that 104 snoRNAs (86 upregulated and 18 downregulated) were differentially expressed in the TP53-mutant subgroup, whereas 59 snoRNAs (23 upregulated and 36 downregulated) were altered in TP53-WT tumors (Table 4) [59]. Among these, 38 snoRNAs (20 upregulated and 18 downregulated) were common to both groups. Importantly, 17 of the 36 members of the HBII-52 cluster were consistently downregulated across both TP53 subgroups, with HBII-52-30 representing the only member not differentially expressed compared to non-tumor pediatric control samples. Additionally, nearly all remaining members (35 of 36) were downregulated specifically in TP53-WT cases (Table 4) [59].

## 5. Vault RNAs

Human vault RNAs (vtRNAs or vRNAs) are typically less than 100 nt in length, with three expressed genes hvg-1, hvg-2, and hvg-3 [29]. Although initially identified as components of vault ribonucleoprotein complexes, only a small fraction of vRNAs associate with the vault complex, pointing to broader regulatory roles in cellular processes like apoptosis and drug resistance [62,63]. In vitro studies have shown that human glioblastoma-derived cell line U118MG exhibits reduced vRNA expression compared to other cancer cell lines and heightened sensitivity to the chemotherapeutic drug mitoxantrone (Table 1) [29]. Interestingly, suppressing vRNA expression through RNA interference progressively reduced mitoxantrone resistance, while overexpression of vRNA-1 increased resistance. Together, these findings suggest that vRNA contributes to mitoxantrone resistance in malignant cells and highlights the potential importance of vRNAs as targets or therapeutic tools in cancer treatment.

## 6. snRNAs

Small nuclear RNAs (snRNAs) are generally less than 200 nucleotides in length and function primarily in pre-mRNA processing [64]. In humans, 10 snRNA gene families have been identified, namely, U1, U2, U4, U5, U6, U7, U4atac, U6atac, U11, and U12 [65]. Most snRNAs function as components of small nuclear ribonucleoproteins (snRNPs) within the spliceosome and contribute to the recognition of splice sites and removal of introns from pre-mRNA. The major spliceosome comprises five core snRNAs, U1, U2, U4, U5, and U6, which function cooperatively in the splicing of the majority of introns [64]. In contrast, U11, U12, U4atac, and U6atac, together with U5, form the minor spliceosome, which processes the less abundant class of U12-type introns [66]. U7 snRNA has a distinct function in the 3′ end processing of replication-dependent histone pre-mRNAs rather than in pre-mRNA splicing [66].

Three snRNAs were reported to be overexpressed in pediatric diffuse intrinsic pontine glioma (DIPG) compared with control tissues, with no downregulated snRNAs identified; however, the specific snRNAs were not reported (Table 1) [20].

In a separate study, U6 snRNA, has been reported to be significantly enriched in serum-derived microvesicles from patients with glioblastoma compared with healthy controls and was identified as an independent predictor of glioblastoma diagnosis (Table 1) [22]. Another study reported that U6 levels were elevated in glioma cell lines compared to normal neuroglial cells, including astrocytes and neuroprogenitors, although no statistical testing was performed for this comparison. (Table 1) [21]. The authors proposed that this difference may be related to the enhanced transcriptional and splicing activity characteristic of cancer cells [21]. Mechanistic studies in the same report identified U6 snRNA as a major target of the oncogenic miRNA miR-10b in glioblastoma. Using CLEAR-CLIP and complementary biochemical and imaging approaches, the authors demonstrated direct miR-10b–U6 interactions in glioma cells and patient-derived GBM tumors and showed that miR-10b modulates U6 stability, conformation, post-transcriptional modifications, and interactions with the spliceosomal proteins SART3 and PRPF8 [21]. These alterations were associated with changes in alternative splicing, including regulation of CDC42 isoforms, and effects on glioma cell viability [21]. Together, these findings provide mechanistic evidence for a role of U6 in miR-10b-mediated regulation of splicing in glioma but do not by themselves establish U6 as a clinically validated diagnostic or prognostic biomarker.

## 7. Mt-tRNAs

Mitochondrial tRNAs (mt-tRNAs) are transfer RNA molecules found within the mitochondria. They are encoded by mtDNA and help to translate the proteins encoded by mitochondrial genome [67]. Recent studies have revealed that post-transcriptional modifications of mt-tRNAs are key modulators of mitochondrial function and metabolic adaptation in GSCs and GICs [68]. The mitochondrial methyltransferase METTL8, for example, is upregulated in GSCs and glioblastoma tissue [68,69]. METTL8 catalyzes the 3-methylcytidine (m^3^C) modification on specific mt-tRNAs, including mt-tRNA^Thr^ and mt-tRNA^Ser^ [68]. METTL8-mediated m^3^C modification increased mitochondrial translation and mitochondrial respiration, highlighting its essential role in maintaining the elevated oxidative phosphorylation (OXPHOS) activity that characterizes GSCs [68]. This METTL8-driven mt-tRNA modification is a mechanism by which GSCs may sustain their bioenergetic demands and tumorigenic potential. Although METTL8 overexpression is a consistent feature of gliomas, its association with patient survival remains unclear. The study by Lee et al. reports that elevated METTL8 levels were correlated with inferior glioma outcomes across multiple cohorts [68]. However, the Schöller et al. study, using only one cohort, did not identify any significant association between METTL8 expression and overall survival in glioblastoma or low-grade glioma patients [69].

In addition to METTL8, the mitochondrial enzyme cyclin-dependent kinase 5 regulatory subunit associated-protein 1 (CDK5RAP1) has been identified as crucial for the maintenance of glioblastoma initiating cells (GICs). CDK5RAP1 facilitates methylthiolation at position 37 of mt-tRNAs, converting isopentenyladenosine (i^6^A) to 2-methylthio-isopentenyladenosine (ms^2^i^6^A) [70]. This modification is essential for preserving GIC-related traits, including self-renewal capacity, undifferentiated status, and tumorigenic potential, independent of mitochondrial protein translation control [70]. Immunofluorescence studies confirm the mitochondrial localization of ms^2^i^6^A-modified tRNAs within GICs [71]. The activity of CDK5RAP1 supports tumor cell survival by detoxifying endogenous i^6^A, thereby preventing excessive autophagy that would otherwise impair tumor maintenance [71]. These findings suggest that CDK5RAP1-mediated mt-tRNA modifications represent a vital adaptive mechanism exploited by glioma cells to sustain malignancy.

## 8. Discussion

Gliomas, particularly glioblastoma, remain among the most therapeutically challenging malignancies due to their molecular heterogeneity, adaptive phenotype, and resistance to conventional therapies. Non-coding RNAs, once considered incidental transcriptional byproducts, are increasingly recognized as active regulators of glioma biology. The evidence summarized in this review indicates that small and intermediate-length ncRNAs are not passive bystanders in this process but active participants in several of the pathways that define malignant behavior and that a deeper mechanistic understanding of their roles may aid in the development of minimally invasive diagnostics and novel therapeutic targets.

The differential upregulation and downregulation of small and intermediate-size ncRNAs in low-grade and high-grade gliomas relative to non-tumor tissue suggests that individual transcripts can either act as tumor suppressors that constrain progression or as oncogenic drivers that promote malignancy, depending on the specific RNA and cellular context. Distinct clusters of snoRNAs were significantly increased in high-risk low-grade glioma, for example, while others were consistently downregulated in glioma tissue relative to controls (Table 2 and Table 3), and similarly divergent patterns were seen among tRNAs and trFs across glioma subtypes (Table 1). A recurring mechanistic theme across these classes is that dysregulation of a given ncRNA is frequently coupled to altered activity of the enzyme responsible for its biogenesis or chemical modification: TRMT10A, METTL1, PUS7, YRDC, DKC1, METTL8, CDK5RAP1, and NSUN6 all sit at this interface, and several of these enzymes are themselves overexpressed in glioma tissue and have been associated tumor with grade or survival [19,22,23,26,28,54,67,71]. Methylation, in particular, can enhance tRNA stability by shielding transcripts from endonucleolytic cleavage, increasing their availability for translation while at the same time reducing the likelihood that a modified tRNA will be processed into a regulatory tRF [18]. The functional consequence of this shift toward stabilized, intact tRNA appears to depend on the specific enzyme and RNA substrate rather than following a single direction, however. In breast cancer, for example, NSUN2-mediated 5mC methylation of tRNA-Val-CAC drives codon-biased translation that supports glycolysis and tumor progression [72,73]. In glioma, by contrast, NSUN6-mediated 5mC modification of mt-tRNAs is associated with a survival benefit in glioblastoma patients and with altered response to temozolomide, rather than with enhanced tumor growth [71]. This divergence suggests that the role of a given RNA modification in glioma should not be assumed from its role in other cancer types without direct evidence and that the modifying enzymes themselves, rather than the modification in isolation, may be the more generalizable point of biological and therapeutic interest.

Because they are stable and readily detectable outside the tumor microenvironment, ncRNAs have attracted particular attention as diagnostic and prognostic biomarkers in bodily fluids such as CSF, blood, saliva, and urine. Within the classes reviewed here, tRNA-derived fragments stand out most clearly: their consistent detection in CSF and plasma and specifically their disproportionate representation among differentially expressed cell-free RNAs in CSF position them among the more immediately translatable candidates for liquid biopsy-based glioma detection [36,37]. U6 snRNA has similarly been detected at significantly higher levels in serum-derived microvesicles from glioblastoma patients than from healthy controls, where it functions as an independent predictor of diagnosis [64]. Together, these findings support the broader premise that ncRNA detection in circulating extracellular vesicles or cell-free fractions could complement, and eventually reduce reliance on, tissue sampling and conventional imaging. Realizing this potential in the clinic, however, will require standardized detection platforms, large-scale validation studies, and a clearer understanding of how individual ncRNA signatures correlate with clinical outcomes.

snoRNAs represent the largest and most mechanistically developed body of evidence reviewed here. Multiple prognostic signatures have been derived independently in low-grade glioma cohorts, and individual snoRNAs such as SNORD12B, SNORD47, SNORD76, SNORD44, and U3 have been functionally linked to glycolipid metabolism, glycolysis, and ribosome biogenesis in cellular models [43,44,46,47,50]. The pediatric data adds an important dimension: distinct snoRNA deregulation patterns by H3F3A and TP53 mutation status, and the consistent downregulation of the HBII-52/SNORD115 cluster in pediatric high-grade glioma, suggest that snoRNA dysregulation is shaped by the underlying driver mutation rather than being a uniform feature of malignant transformation [55]. This raises the possibility that snoRNA profiles could eventually help distinguish molecular subtypes in addition to serving as prognostic markers.

Vault RNAs and snRNAs remain comparatively underexplored, but the available evidence links both to clinically relevant processes. Reduced vRNA expression sensitizes the U118MG glioblastoma line to the chemotherapeutic agent mitoxantrone, while overexpression of vRNA-1 increases resistance, implicating vRNA in chemoresistance rather than in proliferation or glioma stem cell maintenance per se [58]. This raises the untested possibility that vRNA could similarly modulate sensitivity to agents used in standard glioma regimens, such as temozolomide. Small nuclear RNAs illustrate a complementary mechanism: the interaction between the oncogenic microRNA miR-10b and U6 snRNA alters U6’s stability, post-transcriptional modification, and engagement with the splicing factors SART3 and PRPF8, with downstream effects on cell viability [65]. These findings suggest that ncRNAs beyond the well-studied miRNAs, lncRNAs, and circRNAs can influence both drug resistance and splicing regulation and may therefore warrant consideration as therapeutic targets or predictors of treatment response.

Mitochondrial tRNA modifications add a further layer, connecting the epitranscriptome to mitochondrial bioenergetics. METTL8-mediated m3C modification and CDK5RAP1-mediated methylthiolation of mt-tRNAs both sustain the elevated oxidative phosphorylation and self-renewal capacity characteristic of glioma stem-like cells [67,69]. Together with the NSUN6 findings discussed above, this indicates that mitochondrial RNA modification pathways may influence both tumor bioenergetics and treatment response through mechanisms distinct from DNA methylation markers, such as MGMT promoter status, currently used in clinical practice.

Despite this progress, research on small and intermediate-length ncRNAs in glioma remains at an early stage. Most existing studies are descriptive, reporting differential expression or post-transcriptional modification without fully elucidating the mechanisms that drive glioma progression, and few findings have been replicated in independent, multi-institutional cohorts. This limitation is not confined to single-institution cohorts: large public repositories such as TCGA, while enabling analyses across hundreds of patients, consist of tissue specimens collected retrospectively across contributing centers, with limited prospective clinical follow-up. For the single-institution cohorts discussed above, published methods sections typically report ethics approval and informed consent but do not specify whether tissue accrual itself was prospective or retrospective. Regardless of accrual design, however, none of these studies were structured as prospective trials intended to validate a signature’s predictive performance against predefined clinical endpoints in an independent cohort.

Furthermore, nomenclature for tRFs, snRNAs and snoRNAs is also inconsistent across studies, complicating cross-study comparison, and statistical rigor varies: some reports describe expression differences without formal statistical testing. Most functional work, moreover, relies on in vitro glioma cell lines or xenograft models, with limited validation in immunocompetent in vivo systems. Because pediatric and adult gliomas are molecularly distinct, ncRNA signatures identified in one population should also not be assumed to generalize to the other.

The scope of this review itself is a limitation worth acknowledging. Several additional small RNA species, including Y RNAs, 5S and 5.8S rRNA, signal recognition particle (SRP) RNA, and mitochondrial mRNA and rRNA, have established roles in cancer biology more broadly but are not addressed in the sections above because glioma-specific evidence for them remains minimal. For most of these species, only a single study, if any, has examined their expression in glioma, often without formal statistical testing or with inconclusive results. Whether these ncRNA classes contribute meaningfully to glioma pathogenesis, and whether they hold diagnostic or therapeutic value comparable to the classes reviewed here, remain open questions.

Advancing this body of work toward clinical application will require larger, prospectively collected cohorts studied on harmonized sequencing platforms; functional validation of candidate ncRNAs and their modifying enzymes in vivo; and assay standardization for biofluid-based detection, particularly for CSF- and plasma-derived tRFs and snoRNAs, where analytical variability between studies remains a barrier to translation. Combining small and intermediate-length ncRNAs with the more established miRNA, lncRNA, and circRNA biomarkers into multi-analyte panels may also improve diagnostic and prognostic performance beyond what any single ncRNA class can offer. A growing understanding of ncRNA biology may likewise inform the development of ncRNA-targeted therapeutics, and given the apparent role upstream of several dysregulated ncRNAs, the modifying enzymes described here warrant dedicated investigation as potential targets for inhibitors or modulators, an avenue that remains largely unexplored in glioma.

## 9. Conclusions

Small and intermediate-length ncRNAs, including tRNAs and their derived fragments, snoRNAs, vault RNAs, snRNAs and mitochondrial tRNAs represent an emerging and underexplored class of regulatory molecules with significant relevance to glioma biology. The evidence summarized in this review highlights their diverse expression patterns and functional involvement in tumorigenesis, progression, metabolism, immune regulation, and therapeutic response, extending the ncRNA landscape beyond the miRNAs, lncRNAs, and circRNAs that have dominated previous reviews. Their differential expression across tumor grade, molecular subtype, and patient outcome, together with their detectability in tumor tissue and circulating biofluids, such as plasma and CSF, position these ncRNAs as promising candidates for minimally invasive diagnostic and prognostic biomarkers. To date, none of the prognostic or diagnostic associations discussed in this review have been evaluated in a prospective trial designed to validate their clinical performance against predefined endpoints in an independent cohort. This holds whether the underlying evidence derives from TCGA, where tissue collection was retrospective, or from single-institution cohorts, whose accrual design is not consistently reported in the published methods.

Beyond their potential as biomarkers, emerging evidence suggests that certain small and intermediate-length ncRNAs and the enzymes responsible for their epitranscriptomic modifications may represent potential therapeutic targets. However, current studies remain largely focused on expression profiling and descriptive associations, and substantial gaps remain in understanding the molecular mechanisms through which these ncRNAs influence glioma development and treatment response. Prospectively designed clinical cohorts, standardized detection and profiling methods, and mechanistic studies will therefore be essential to establish their clinical significance. As RNA profiling and therapeutic technologies continue to advance, integrating these and other understudied small and intermediate-length ncRNAs into existing molecular frameworks may provide new opportunities for precision medicine and ultimately contribute to improved diagnosis, prognosis, and treatment of gliomas.

## Figures and Tables

**Table 1:** Non-coding RNAs altered in gliomas.

RNA Name	Expression Pattern in Glioma vs. Normal	LGG vs. GB
**snRNA**	↑ in pediatric DIPG vs. control tissues [20]	—
**U6 (RNU6-1)**	Appears ↑ in glioma cells vs. normal neuroglia cells (no statistical testing performed) [21] ↑ in serum-derived microvesicles from glioblastoma patients vs. healthy controls [22]	—
**tRNAeMet**	—	↑ in GB vs. grade II/III tissue [23]
**tRNAiMet**	—	↑ in GB vs. grade II/III tissue [23]
**LeuCAA fragment**	↑ in plasma-derived exosomes of glioma patients compared to healthy controls [24]	—
**tiRNA-1-33-Gly-GCC-1**	—	↑ in GB vs. LGG [25]
**tiRNA-1-33-Gly-GCC-2-M3**	—	↑ in GB vs. LGG [25]
**tiRNA-1-34-Lys-CTT-1-M2**	—	↑ in GB vs. LGG [25]
**tiRNA-1-34-Lys-CTT-3**	—	NSD between GB and LGG [25]
**tRFdb-3003a**	↓ in LGG astrocytoma tissues compared to non-tumor controls [26]	—
**tRFdb-3003b**	↓ in LGG astrocytoma tissues compared to non-tumor controls [26]	—
**tRFdb-5017a**	NSD between LGG astrocytoma tissues and non-tumor controls [26]	—
**tRFdb-5017b**	NSD between LGG astrocytoma tissues and non-tumor controls [26]	—
**tRF-+1-T17-Ser-TGA-1**	—	NSD between GB and LGG [25]
**tRF-1-22-chrM.Ser-GCT**	—	NSD between GB and LGG [25]
**tRF-1-31-His-GTG-1**	—	↑ in GB vs. LGG [25]
**tRF-1-32-chrM.Lys-TTT**	—	↓ in GB vs. LGG [25]
**tRF-16-Y9H33PD**	↑ in CSF of glioma patients compared to normal CSF [27]	—
**tRF-16-8R6546D**	NSD in plasma from glioma patients compared to normal plasma [27]	—
**tRF-17-8SOUPR2**	↓ in CSF of glioma patients compared to normal CSF [27]	—
**tRF-18-HRMFWRV**	NSD in plasma from glioma patients and plasma from control patients [27]	—
**tRF-20-R959KUMK**	↑ in CSF of glioma patients compared to normal CSF [27]	—
**tRF-22-R3WE8RO84**	↑ in CSF of glioma patients compared to normal CSF [27]	—
**tRF-26-R3WE8RO86JD**	NSD in plasma from glioma patients and plasma from control patients [27]	—
**tRF-26-R38E8RO86JD**	↑ in plasma of glioma patients compared to plasma from control patients [27]	—
**tRF-26-3K7SIR3DR20**	↑ in CSF of glioma patients compared to normal CSF [27]	—
**tRF-28-08R959KUMK9**	NSD in plasma from glioma patients and plasma from control patients [27]	—
**tRF-30-F1R3WE8RO86J**	↑ in CSF of glioma patients compared to normal CSF [27]	—
**tRF-30-PNR8YP9LON4V**	↑ in U87, U251 and U373 glioma cells compared with the human astrocytes [28]	NSD between LGG and GB [28]
**tRF-30-87R8WP9N1EWJ**	↑ in U87, U251 and U373 glioma cells compared with human astrocytes [28]	NSD between LGG and GB [28]
**tRF-32-FZ9KR959NRZZP**	↑ in CSF of glioma patients compared to normal CSF [27]	—
**tRF-34-M2OSRNLNKSEKH9**	↑ in CSF of glioma patients compared to normal CSF [27]	—
**tRF-35-DEU2O1B54ZUPX1**	NSD in plasma from glioma patients and plasma from control patients [27]	—
**tRF-35-QKF1R3WE8RO86J**	↑ in CSF of glioma patients compared to normal CSF [27]	—
**tRF-37-QB1MK8YUBS68BFJ**	↑ in CSF of glioma patients compared to normal CSF [27]	—
**tRF-39-J9ZH0SR9593KMHFV**	↑ in CSF of glioma patients compared to normal CSF [27]	—
**ts-55**	↓ in LGG astrocytoma tissues compared to non-tumor controls [26]	—
**ts-60**	↓ in LGG astrocytoma tissues compared to non-tumor controls [26]	—
**vRNA**	↓ in glioma cell line U118MG and more sensitive to mitoxantrone compared to other cancers types [29]	—

GB: glioblastoma, LGG: low-grade glioma, CSF: cerebrospinal fluid, ↑: upregulated, ↓: downregulated, NSD: not significantly different, —: unknown.

**Table 2:** snoRNAs altered in gliomas.

snoRNA	Expression in Glioma vs. Normal	LGG vs. HGG	Role	Functional Association
**h-jouvence (h-jou)**	↓ in U87 glioblastoma cells compared to non-glioma cell lines [47]	—	Potential oncogenic driver	Low expression in U87 cell line→ decreased proliferation, associated with apoptosis; overexpression in cellular model stimulates cell proliferation; knockdown of h-jou decreases cancerous cell proliferation
**SCARNA4**	↑ in pediatric DIPG vs. control tissue [20]	—	Potential Biomarker	—
**SCARNA8**	↑ in pediatric DIPG vs. control tissue [20]	—	Potential Biomarker	—
**SNORA10**	↑ in pediatric DIPG vs. control tissue [20]	—	Potential Biomarker	—
**SNORA11E**	—	↑ in DIPG vs. pediatric non-brainstem astrocytoma [20]	Potential Biomarker	—
**SNORA2A**	↑ in pediatric DIPG vs. control tissue [20]	—	Potential Biomarker	—
**SNORA31**	↑ in pediatric DIPG vs. control tissue [20]	—	Potential Biomarker	—
**SNORA38B**	↑ in pediatric DIPG vs. control tissue [20]	—	Potential Biomarker	—
**SNORA54**	↑ in pediatric DIPG vs. control tissue [20]	↑ in DIPG vs. pediatric non-brainstem astrocytoma [20]	Potential Biomarker	—
**SNORA71B**	↑ in glioma vs. normal brain tissue [48]	↑ in GB vs. LGG tissue [48]	Potential oncogenic driver	Higher expression in tissue → higher grade, overall survival and disease-free survival were negatively correlated with SNORA71B expression levels in retrospective transcriptomic analysis; knockout of *SNORA71B* decreased cell viability migration, and invasion [48]
**SNORD12B**	↑ in glioma vs. normal brain tissue and glioblastoma cell lines vs. normal human astrocytes [49]	↑ in WHO III-IV vs. WHO I-II tissue [49]	Potential oncogenic driver	Higher expression in tissue → higher grade in retrospective transcriptomic analysis; knockdown of SNORD12B in cellular models inhibited glioblastoma cell glycolipid metabolism and proliferation
**SNORD38A**	NSD in plasma of glioma patients compared to normal plasma [27]	—	—	—
**SNORD41**	—	↓ in DIPG vs. pediatric non-brainstem astrocytoma [20]	Potential Biomarker	—
**SNORD44**	↓ in glioma tissue and cells [50]	↓ WHO IV-III vs. WHO I-II tissue [50]	Potential Tumor Suppressor	Lower expression in tissue → higher grade in retrospective transcriptomic analysis; overexpression in cellular model suppresses growth, invasion, and migration [50]
**SNORD47 (RNU47/U47)**	↓ in glioma tissue vs. adjacent non-tumor tissue [51]	↓ in WHO IV vs. WHO III and I/II tissue [51]	Potential Tumor Suppressor	Lower expression → higher grade and shorter OS in retrospective transcriptomic analysis; overexpression in cellular model inhibits cell proliferation and colony formation
**SNORD53**	↑ in pediatric DIPG vs. control tissue [20]	—	Potential Biomarker	—
**SNORD76 (U76)**	↓ in glioma WHO grade III and IV tissue vs. non-tumor brain surgically resected tissues [46] ↓ in human GB cell lines vs. normal human astrocytes [52]	↓ in WHO IV vs. WHO II and III resected tissue [53]	Potential Tumor Suppressor	Lower expression → higher grade and shorter OS in retrospective transcriptomic analysis; overexpression in cellular model inhibits cell proliferation and colony formation
**SNORD91B**	—	↓ in DIPG vs. pediatric non-brainstem astrocytoma [20]	Potential Biomarker	—
**SNORD92**	↑ in pediatric DIPG vs. control tissue [20]	—	Potential Biomarker	—
**SNORD104**	↓ in plasma of glioma patients compared to normal plasma [27]	—	Potential Biomarker	Lower expression in plasma → glioma
**SNORD113-7**	—	↑ in DIPG vs. pediatric non-brainstem astrocytoma [20]	Potential Biomarker	—
**SNORD114-5**	—	↑ in DIPG vs. pediatric non-brainstem astrocytoma [20]	Potential Biomarker	—
**SNORD115-6**	—	↓ in DIPG vs. pediatric non-brainstem astrocytoma [20]	Potential Biomarker	—
**SNORD115-10**	↓ in pediatric DIPG vs. control tissue [20]	↓ in DIPG vs. pediatric non-brainstem astrocytoma [20]	Potential Biomarker	—
**SNORD115-15**	↓ in pediatric DIPG vs. control tissue [20]	↓ in DIPG vs. pediatric non-brainstem astrocytoma [20]	Potential Biomarker	—
**SNORD115-17**	↓ in pediatric DIPG vs. control tissue [20]	—	Potential Biomarker	—
**SNORD115-18**	↓ in pediatric DIPG vs. control tissue [20]	—	Potential Biomarker	—
**SNORD115-19**	↓ in pediatric DIPG vs. control tissue [20]	—	Potential Biomarker	—
**SNORD115-22**	—	↓ in DIPG vs. pediatric non-brainstem astrocytoma [20]	Potential Biomarker	—
**SNORD115-24**	↓ in pediatric DIPG vs. control tissue [20]	—	Potential Biomarker	—
**SNORD115-25**	↓ in pediatric DIPG vs. control tissue [20]	—	Potential Biomarker	—
**SNORD115-27**	↓ in pediatric DIPG vs. control tissue [20]	—	Potential Biomarker	—
**SNORD115-29**	—	↓ in DIPG vs. pediatric non-brainstem astrocytoma [20]	Potential Biomarker	—
**SNORD115-30**	↓ in pediatric DIPG vs. control tissue [20]	—	Potential Biomarker	—
**SNORD115-31**	↓ in pediatric DIPG vs. control tissue [20]	—	Potential Biomarker	—
**SNORD115-32**	↓ in pediatric DIPG vs. control tissue [20]	—	Potential Biomarker	—
**SNORD115-36**	—	↓ in DIPG vs. pediatric non-brainstem astrocytoma [20]	Potential Biomarker	—
**SNORD115-42**	—	↓ in DIPG vs. pediatric non-brainstem astrocytoma [20]	Potential Biomarker	—
**SNORD115-44**	↓ in pediatric DIPG vs. control tissue [20]	—	Potential Biomarker	—
**U3 (RNU3)**	↑ in GB and LGG vs. normal brain tissue and ↑ in IDH1^WT^ vs. IDH1^R132H^ [54]	No statistical comparison between GB and LGG tissues [50]	Potential oncogenic driver	Higher expression in tissue → IDH1^WT^ vs. IDH1^R132H^; Promotes glycolysis and proliferation (U3/ZBTB7A/HK2 LDHA axis); enhances 18S rRNA processing; knockdown of U3 suppresses aerobic glycolysis and proliferation

GB: glioblastoma, LGG: low-grade glioma, DIPG: Diffuse Intrinsic Pontine Glioma, CSF: cerebrospinal fluid, ↑: upregulated, ↓: downregulated, NSD: not significantly different, —: unknown.

**Table 3:** snoRNAs altered in high-risk vs. low-risk LGG tissue.

snoRNA	Expression in High-Risk vs. Low-Risk LGG	Functional Association
**ACA59**	↑ in high-risk LGG [57]	High expression in tissue → associated with shorter OS; potential pro-tumorigenic driver
**SCARNA15**	↑ in high-risk LGG [56]	Higher expression in tissue → associated with poor prognosis; potential pro-tumorigenic driver
**SNORA22B**	↑ in high-risk LGG [57]	High expression in tissue → associated with poor prognosis; potential pro-tumorigenic driver
**SNORA31**	↓ in high-risk LGG [57]	Lower expression in tissue → associated with poor prognosis; potential tumor suppressor
**SNORA32**	↑ in high-risk LGG [56]	Higher expression in tissue → associated with poor prognosis; potential pro-tumorigenic driver
**SNORA36B**	↑ in high-risk LGG [56]	Higher expression in tissue → associated with poor prognosis; potential pro-tumorigenic driver
**SNORA40**	↓ in high-risk LGG [57]	Lower expression in tissue → associated with shorter OS; potential tumor suppressor
**SNORA63**	↑ in high-risk LGG [57]	High expression in tissue → associated with shorter OS; potential pro-tumorigenic driver
**SNORA63E**	↓ in high-risk LGG [57]	Lower expression in tissue → associated with poor prognosis; potential tumor suppressor
**SNORA63D**	NSD [56]	No significant change between high-risk and low-risk LGG
**SNORA72**	↑ in high-risk LGG [57]	High expression in tissue → associated with shorter OS; potential pro-tumorigenic driver
**SNORA80D**	↓ in high-risk LGG [57]	Lower expression in tissue → associated with shorter OS; potential tumor suppressor
**SNORD38A**	↑ in high-risk LGG [56]	Higher expression in tissue → associated with poor prognosis; potential pro-tumorigenic driver
**SNORD73B**	↓ in high-risk LGG [57]	Lower expression in tissue → associated with shorter OS; potential tumor suppressor
**SNORD88C**	↑ in high-risk LGG [56]	Higher expression in tissue → associated with poor prognosis; promotes proliferation, invasion, migration; potential pro-tumorigenic driver
**SNORD91A**	↑ in high-risk LGG [57]	High expression in tissue → associated with poor prognosis; potential pro-tumorigenic driver
**SNORD115-45**	↓ in high-risk LGG [57]	Lower expression in tissue → associated with shorter OS; potential tumor suppressor
**U3**	↓ in high-risk LGG [57]	Lower expression in tissue → associated with shorter OS; potential tumor suppressor

LGG: low-grade glioma, ↑: upregulated, ↓: downregulated, NSD: not significantly different.

**Table 4:** snoRNA expression profiles in pediatric high-grade glioma (pHGG; ≤18 years) brain tissue, stratified by H3F3A and TP53 mutation status and compared with pediatric non-tumor control brain samples. Adapted from [59].

SnoRNA Name (Aliases)	pHGG vs. Control	H3F3A Mutant	H3F3A WT	TP53 Mutant	TP53 WT
**SNORD114-20 (14qII-20)**	Down	Down	Down	Down	Down
**SNORD115-1 (HBII-52-1)**	Down	—	Down	Down	Down
**SNORD115-3 (HBII-52-3)**	Down	—	Down	Down	Down
**SNORD115-5 (HBII-52-5)**	Down	—	Down	Down	Down
**SNORD115-6 (HBII-52-6)**	Down	—	—	—	Down
**SNORD115-7 (HBII-52-7)**	Down	—	Down	—	Down
**SNORD115-8 (HBII-52-8)**	Down	—	Down	Down	Down
**SNORD115-9 (HBII-52-9)**	Down	—	Down	Down	Down
**SNORD115-10 (HBII-52-10)**	Down	—	Down	—	Down
**SNORD115-11 (HBII-52-11)**	Down	—	Down	Down	Down
**SNORD115-12 (HBII-52-12)**	Down	—	Down	Down	Down
**SNORD115-13 (HBII-52-13)**	Down	—	Down	—	Down
**SNORD115-15 (HBII-52-15)**	Down	—	Down	—	Down
**SNORD115-16 (HBII-52-16)**	Down	—	Down	—	Down
**SNORD115-17 (HBII-52-17)**	Down	—	Down	—	Down
**SNORD115-18 (HBII-52-18)**	Down	Down	Down	Down	Down
**SNORD115-19 (HBII-52-19)**	Down	—	Down	—	Down
**SNORD115-20 (HBII-52-20)**	Down	—	Down	Down	Down
**SNORD115-21 (HBII-52-21)**	Down	—	Down	—	Down
**SNORD115-22 (HBII-52-22)**	Down	—	Down	Down	Down
**SNORD115-23 (HBII-52-23)**	Down	—	Down	—	Down
**SNORD115-25 (HBII-52-25)**	Down	—	Down	—	Down
**SNORD115-26 (HBII-52-26)**	Down	—	Down	Down	Down
**SNORD115-27 (HBII-52-27)** **(ENSG00000201300)**	Down	—	Down	Down	Down
**SNORD115-29 (HBII-52-29)**	Down	—	Down	—	Down
**SNORD115-30 (HBII-52-30)**	Down	—	Down	—	—
**SNORD115-31 (HBII-52-31)**	Down	—	Down	Down	Down
**SNORD115-32 (HBII-52-32)**	Down	—	Down	—	Down
**SNORD115-33 (HBII-52-33)**	Down	—	Down	Down	Down
**SNORD115-36 (HBII-52-36)**	Down	—	Down	—	Down
**SNORD115-38 (HBII-52-38)**	Down	—	Down	Down	Down
**SNORD115-40 (HBII-52-40)**	Down	—	Down	—	Down
**SNORD115-41 (HBII-52-41)**	Down	—	Down	Down	Down
**SNORD115-42 (HBII-52-42)**	Down	—	Down	—	Down
**SNORD115-43 (HBII-52-43)**	Down	—	Down	Down	Down
**SNORD115-44 (HBII-52-44)**	Down	—	Down	—	Down
**SNORD115-48 (HBII-52-48)**	Down	—	Down	—	Down
**ENSG00000238956**	Up	Up	Up	Up	—
**ENSG00000252543**	Up	—	Up	Up	Up
**SCARNA11 (ACA57)**	Up	—	Up	Up	—
**SCARNA13 (U93)**	Up	—	Up	Up	—
**SNORA7 (ENSG00000206913)**	Up	—	Up	Up	—
**SNORA7A (ACA7)**	Up	—	Up	Up	—
**SNORA7B (ACA7B)**	Up	—	Up	Up	—
**SNORA8 (ACA8)**	Up	Up	—	Up	—
**SNORA10 (ACA10)**	Up	—	—	Up	—
**SNORA10B (ENSG00000207187)**	Up	—	Up	Up	—
**SNORA15 (ACA15)**	Up	—	Up	Up	—
**SNORA15B-1 (ENSG00000207062)**	Up	—	Up	Up	—
**SNORA15B-2 (ENSG00000206785)**	Up	—	Up	Up	—
**SNORA16A (ACA16)**	Up	Up	Up	Up	—
**SNORA22B (ENSG00000206603)**	Up	Up	Up	Up	Up
**SNORA24 (ACA24** or **ENSG00000275994)**	Up	Up	Up	Up	—
**SNORA24 (SNORA24L2** or **ENSG00000207130)**	Up	Up	—	Up	—
**SNORA24B (ENSG00000206903)**	Up	Up	Up	Up	—
**SNORA38 (ACA38)**	Up	Up	Up	Up	Up
**SNORA38B**	Up	Up	Up	Up	—
**SNORA40 (ACA40)**	Up	Up	Up	Up	Up
**SNORA40B (ENSG00000208308)**	Up	—	—	—	—
**SNORA41 (ACA41)**	Up	Up	Up	Up	—
**SNORA48 (ACA48)**	Up	Up	Up	Up	—
**SNORA51 (ACA51)**	Up	—	Up	Up	—
**SNORA52 (ACA52)**	Up	—	—	Up	—
**SNORA61 (ACA61)**	Up	—	Up	Up	—
**SNORA67 (U67)**	Up	Up	Up	Up	Up
**SNORA68 (U68)**	Up	Up	Up	Up	—
**SNORA73B (U17B)**	Up	Up	—	Up	—
**SNORA75 (U23)**	Up	—	Up	Up	—
**SNORA79B (ENSG00000222489)**	Up	—	Up	Up	—
**SNORA80E (ACA42)**	Up	—	—	—	—
**SNORA84**	Up	—	—	—	—
**SNORD1B (snR38B)**	Up	Up	—	Up	—
**SNORD1C (snR38C)**	Up	Up	Up	Up	Up
**SNORD2 (snR39B)**	Up	Up	Up	Up	—
**SNORD4A (mgh18S-121)**	Up	Up	—	Up	—
**SNORD5 (mgh28S-2409)**	Up	Up	—	Up	—
**SNORD6A (mgh28S-2411)**	Up	Up	Up	Up	—
**SNORD12B (HBII-99B)**	Up	Up	—	Up	—
**SNORD13 (U13)**	Up	—	Up	Up	—
**SNORD14B (U14B)**	Up	Up	—	Up	—
**SNORD19C (ENSG00000222345)**	Up	Up	—	—	Up
**SNORD24 (U24)**	Up	Up	Up	Up	Up
**SNORD25 (U25)**	Up	Up	Up	Up	—
**SNORD26 (U26)**	Up	Up	Up	Up	Up
**SNORD27 (U27)**	Up	Up	—	Up	—
**SNORD28 (U28)**	Up	Up	Up	Up	—
**SNORD30 (U30)**	Up	Up	Up	Up	—
**SNORD31 (U31)**	Up	Up	Up	Up	Up
**SNORD33 (U33)**	Up	Up	Up	Up	—
**SNORD34 (U34)**	Up	Up	Up	Up	—
**SNORD35A (U35A)**	Up	Up	Up	Up	—
**SNORD36A (U36A)**	Up	Up	Up	Up	—
**SNORD37 (U37)**	Up	—	Up	Up	Up
**SNORD38A (U38A)**	Up	Up	Up	Up	Up
**SNORD38B (U38B)**	Up	—	Up	Up	—
**SNORD41 (U41)**	Up	Up	Up	Up	—
**SNORD42A (U42 or U42A)**	Up	Up	Up	Up	—
**SNORD42B (U42B)**	Up	Up	—	Up	—
**SNORD46 (U46)**	Up	Up	—	Up	—
**SNORD48 (U48)**	Up	Up	Up	Up	—
**SNORD51 (U51)**	Up	Up	Up	Up	Up
**SNORD53 (U53)**	Up	—	Up	Up	—
**SNORD55 (U55)**	Up	Up	Up	Up	—
**SNORD56 (U56)**	Up	Up	Up	Up	Up
**SNORD57 (U57)**	Up	Up	Up	Up	—
**SNORD58 (U58C)**	Up	Up	—	Up	—
**SNORD62 (ENSG00000199411)**	Up	—	Up	Up	—
**SNORD69 (HBII-210)**	Up	Up	Up	Up	—
**SNORD74 (U74)**	Up	Up	Up	Up	Up
**SNORD75 (U75)**	Up	Up	Up	Up	—
**SNORD76 (U76)**	Up	Up	Up	Up	Up
**SNORD77 (U77)**	Up	Up	—	—	Up
**SNORD78 (ENSG00000212378)**	Up	Up	Up	Up	Up
**SNORD78 (U78)**	Up	Up	Up	Up	Up
**SNORD79 (U79)**	Up	Up	—	Up	—
**SNORD82 (U82)**	Up	Up	Up	Up	—
**SNORD83A (U83A)**	Up	Up	Up	Up	Up
**SNORD83B (U83B)**	Up	Up	Up	Up	Up
**SNORD88A (HBII-180A)**	Up	Up	Up	Up	—
**SNORD88C (HBII-180C)**	Up	Up	Up	Up	—
**SNORD93 (HBII-336)**	Up	—	—	Up	—
**SNORD97 (U97)**	Up	Up	Up	Up	—
**SNORD99 (HBII-420)**	Up	—	Up	Up	—
**SNORD116 (ENSG00000202498)**	Up	—	Up	Up	Up
**SNORD116-6 (HBII-85-6)**	Up	—	—	—	—
**SNORD116-26 (HBII-85-26)**	Up	—	Up	—	—
**SNORD117 (U83)**	Up	Up	—	Up	—
**SNORD118 (U8)**	Up	Up	—	Up	—
**SNORD119**	Up	Up	Up	Up	—
**SNORD125**	Up	—	—	—	Up

Up: upregulated, Down: downregulated —: not differentially expressed.

## Data Availability

No new data were created or analyzed in this study. Data sharing is not applicable.
